# Adolescent alcohol consumption: protocol for a scoping review of screening and assessment tools used in Africa

**DOI:** 10.1186/s13643-021-01653-1

**Published:** 2021-04-08

**Authors:** Eleanor Briegal, Alice M. Biggane, Angela I. Obasi

**Affiliations:** 1 Department of International Public Health, The Liverpool School of Tropical Medicine, Liverpool, UK; 2 AXESS Sexual Health, Liverpool University Hospitals NHS Foundation Trust, Liverpool, UK

**Keywords:** Scoping review, Alcohol screening tools, Alcohol assessment tools, Adolescents, Africa

## Abstract

**Background:**

Alcohol consumption is a key public health challenge in sub-Saharan Africa, which has the highest burden of alcohol attributable injury and disease of any region. Excess alcohol use is particularly harmful for adolescents and has been associated with neurocognitive defects and social and emotional problems. Effective screening and assessment tools are necessary to implement, evaluate and monitor interventions to prevent and decrease adolescent alcohol use. Most of these tools have been used among adolescent groups in high income settings; data on their effectiveness in Africa, where much alcohol use is unregulated, is limited. This scoping review will examine and map the range of tools in use and create an evidence base for future research in adolescent alcohol prevention and control in Africa.

**Methods:**

The review will include all relevant study designs and grey literature. Inclusion and exclusion criteria have been designed using the Population – Concept – Context framework, and two reviewers will independently screen titles, abstracts and then full text to determine eligibility of articles. The Cochrane Library, MEDLINE, CINAHL and Global Health data bases will be searched for peer reviewed publications. The search strategy for grey literature will include Google searches and searches in websites of pertinent professional bodies and charities. The methodological framework proposed by Arksey and O’Malley and adaptations by the Joanna Briggs Institute and Levac et al. will be used. An iterative approach to charting, collating, summarising and reporting the data will be taken, with the development of charting forms and the final presentation of results led by the extracted data.

**Results:**

This scoping review protocol describes a secondary analysis of data already collected to explore and map alcohol consumption measurement tools in adolescents in Africa.

**Conclusions:**

It is anticipated that our findings will provide an evidence base surrounding tools used to measure adolescent alcohol consumption in Africa. These findings are likely to be useful in informing future research, policy and public health strategies. Findings will be disseminated widely through peer-reviewed publication and in various media, for example, conferences, congresses or symposia.

**Systematic review registration:**

Scoping Review Registration: Open Science Framework (https://osf.io/bjhgw/)

**Supplementary Information:**

The online version contains supplementary material available at 10.1186/s13643-021-01653-1.

## Background

With 3 million deaths per year attributable to its use, alcohol is a major public health problem world-wide [1–4]. This is particularly true in sub-Saharan Africa which has the highest burden of disease and injury attributed to alcohol of any region [1, 5]. Alcohol related harms include sexual risk taking [6], adverse HIV outcomes, self-harm, suicide, and the perpetration of sexual violence [7].

Adolescent drinking in particular is a major global health concern [8]. Harmful alcohol use in adolescence causes alterations in attention, verbal learning, visuospatial processing and memory, as well as altered development of grey and white matter of the central nervous system [9]. Alongside the neurocognitive impacts of adolescent alcohol use, there is also evidence that alcohol use affects social and emotional development, including family, peer and sexual relationships, as well as causing emotional changes and mental health problems [10, 11].

Risk of harm increases with volume and frequency of alcohol use. It is therefore necessary to quantify patterns of alcohol use among drinkers if risk of harm is to be assessed. Heavy episodic drinking (HED) is defined as “drinking at least 60 g or more of pure alcohol on at least one occasion in the past 30 days” and is one of the most significant indicators for alcohol related harm [12]. Over half of adolescents who drink in Africa engage in HED [1]. It is also known that the alcohol industry has been expanding its commercial activities in Africa [13, 14] using strategies that target young people and women [7].

To implement, monitor and measure the impact of interventions to tackle alcohol misuse, there must be effective screening and assessment tools. However, measuring alcohol use is challenging in sub-Saharan Africa, especially in contexts where traditional homemade brew represents the majority of alcohol use [15]. Measures for the use of homemade brew are hard to standardise. For example, locals may refer to the amount of brew consumed by the amount of money spent [15], rather than the volume of alcohol contained in the beverage. Standardised locally appropriate monitoring tools are therefore needed.

There are a number of screening and assessment tools in use across the world, which rely on self-reported use. Most common are the CAGE questionnaire [16], AUDIT (Alcohol Use Disorders Identification Test) [17] and its modifications and CRAFFT [18], which is specific to adolescents. There is a large body of literature assessing the effectiveness of such tools across a range of settings. Generally, AUDIT is considered to be a strong screening tool for assessing alcohol use in young people [19] and this has been seen in Africa [20]. CRAFFT has also been shown to have adequate psychometric properties for detecting alcohol use disorders in adolescents [21] and found to have adequate internal consistency and acceptable reliability within a population of South African adolescents [22]. Alcohol biomarkers offer an objective measurement of a person’s alcohol consumption but have limitations in that they are best in determining if a patient is abstinent from alcohol or a very heavy consumer [23], they are expensive to use [24] and are particularly inaccessible in sub-Saharan Africa due to weak labatory infrastructure [25].

While there has been some work toward comparing and validating tools, there has been no synthesis of the available evidence for this population in this setting. This needs to be done because alcohol use disorders are a significant cause of mortality and morbidity; treatments are available and outcomes can be improved by early detection and intervention [26]. Standardised, culturally appropriate tools for the implementation and monitoring of these detection and intervention strategies are therefore needed. In order to address this gap, the current scoping review will examine and map the range of tools in use and create an evidence base for future research in this area.

## Methods

A scoping review was considered to be the appropriate methodology to assess alcohol measurement tools. Scoping reviews are unique in their ability to provide a broad overview of a research area, where very specific research questions have not been identified. It is more useful to summarise and disseminate known evidence, identifying gaps in the existing literature, which may lead to identification of research questions for systematic review [27, 28]. Scoping review methodology is also favourable as a range of sources of information, including grey literature, can be included for review. This protocol development has drawn on elements of the Preferred Reporting Items for Systematic reviews and Meta-Analyses extension for Scoping Reviews (PRISMA-ScR) Checklist [29], which will also be used in reporting our findings. For this protocol, we have followed the Preferred Reporting Items for Systematic Review and Meta-Analysis Protocols (PRISMA-P) 2015 statement as appropriate for this review, which can be found in Additional file 1 (Table 1).

**Table 1:** Population Concept Context (PCC) framework providing an overview of the components and characteristics of the research question

Inclusion criteria: population concept context	
Population	Adolescents (ages 10–19) in Africa.
Concept	Literature with a specific focus and/or statement describing alcohol screening tools and/or alcohol biomarkers.
Context	All study designs, reports, blogs, book chapters, editorials and commentaries from the public health field since 2000. English language only.

This scoping review will draw on the stepwise methodological framework described by Arksey and O’Malley [27], with updates by the Joanna Briggs Institute (JBI) [28] and by Levac et al. [30].

### Methodological framework

#### Stage 1: identifying the research questions

Arksey and O’Malley recommend an iterative process and maintaining a wide approach when determining research questions. Based on the gaps in the literature an initial research question has been developed:

- What is the available evidence about the range of tools used to measure alcohol use within adolescent populations in Africa?

As appropriate for this methodology, this research question may be changed, or new ones added as the authors become more familiar with the literature.

#### Stage 2: identifying relevant studies

This study will search for evidence via a number of different sources. The search strategy will be defined by inclusion criteria developed using the “PCC” mnemonic described by the JBI [28]. This stands for the Population, Concept and Context and is similar to the PICO (population, intervention, comparator and outcome) model used to help define clinical questions.

##### Exclusion criteria

The predefined exclusion are as follows:

- Studies with no concept of interest, e.g. those with no mention of how alcohol consumption was measured
- Duplicate publications
- Protocol only
- Not health-related research
- Published before 2000
- Not used in Africa
- Not English language
- Studies including any participants over the age of 26 years*

*During the development of our inclusion criteria, a preliminary review of abstracts showed that many studies including a large age range did not stratify below age 25 years and therefore provided no useful information about adolescents. We have therefore chosen to limit to studies that focus exclusiviely on 10–25-year olds, excluding any study with older participants in order to focus the review.

##### Definition of “adolescents”

The World Health Organization (WHO) define adolescents as individuals within the 10–19-year age group [31]; however, the literature addressing adolescent health is not consistent in the age ranges used. Therefore, this scoping review will include sources of information with participants up to the age of 25 if those in the 10–19 age group have been included. This will later be stratified by age if possible.

This variability of the age ranges used to define adolescence is illustrated in the example search strategies shown in the Additional files 2, 3, 4, and 5, where different limiters and search terms have been used for the different databases due to varying definitions of adolescence. This is a challenge not only for this scoping review but also for all research within adolescent health, and future research must be done to consolidate a definition.

##### Search strategy

Firstly, a comprehensive search of electronic databases for published literature will be undertaken. This review will use the three-step search strategy proposed by the JBI [28]. The first step is an initial limited search of at least two online databases; this review will use MEDLINE and CINAHL. The results of this search will be analysed to identify relevant text words in the titles and abstracts of pertinent papers, as well as index terms used to describe the articles. This will be used to develop the search strategy across all included databases, with additional input from a health librarian specialist. The included databases will be Cochrane Library, MEDLINE, CINAHL and Global Health. The searches will be limited to sources of information after the year 2000, as AUDIT-C, a variation of AUDIT and one of the seminal alcohol screening tests used in adolescents, was developed in 1998 [17].

The third stage of the search strategy will be searching the reference list of included studies.

The search strategy for grey literature will include websites of pertinent professional bodies, charities and non-governmental organisaitons (NGOs). In country reports and government documents will also be searched if these are accessible. Google searches will also be undertaken.

#### Stage 3: study selection

After the search is undertaken, the titles and abstracts of identified records will be imported into a reference manager for deduplication. The selection of studies will involve two stages of screening. Stage 1 will involve the screening of titles and abstracts by two reviewers (EB and AMB) to determine their eligibility for full-text review based on the a priori inclusion and exclusion criteria. These criteria will be tested on a sample of abstracts before the actual search is conducted to test its robustness. Stage 2 will then have two reviewers independently assess full-text articles for whether they meet the inclusion or exclusion criteria. In the case of any disagreement about inclusion, full-text articles will be reviewed again by both reviewers and discussed. In the event of no agreement being reached, a third member of the research team (AIO) will weigh in until a consensus is reached.

On completion of study selection, an adapted version of the PRISMA flow diagram will be completed to report final numbers, detailing reasons for exclusion at the full-text review stage only.

#### Stage 4: charting the data

This stage of work involves ‘charting’ key items of information obtained from the primary research reports being reviewed [27]. Development of a charting form is an iterative process and it will be refined as full-text articles are screened. A draft charting table is shown in Table 2. As the data charting process continues additional categories will be added accordingly.

**Table 2:** Draft data charting form

Draft data charting form	
Study characteristics	Extracted data
General information	Author(s) Year of publication Publication type: e.g. journal article, editorial, conference abstract, grey literature, reports, charity website, government document Purpose of study: e.g. validation study, comparison study, intervention study Aims of study Study population and sample size Older/younger adolescents (10–14/15–19) Gender, male/female or both Geographical location—country Setting (e.g. school/community/clinic) Methods
Measurement tools	Type of screening/assessment tool e.g. screening test or biomarker Original or modified tool Effectiveness of tool Acceptability of tool Tool operator role/skill level

#### Stage 5: collating, summarising and reporting the results

As a scoping study, the purpose is to present an overview of all the material reviewed. The results of the scoping will be presented in the most appropriate way to answer the research question(s) and will be led by the extracted data. It may be useful to categorise results by emerging conceptual categories. This is where gaps in the literature will be identified and if appropriate, suggestions for future systematic review will be highlighted. We plan to analyse the data using descriptive statistics via Microsoft Excel and report the findings narratively. However, we will be adaptive to the data we extract and the subsequent analysis as appropriate. It should be noted that this study will not assess the quality of evidence and therefore cannot comment on the generalisability and robustness of individual studies [27].

##### Data management

We will record our progress and review process thoroughly using secure shared drives between the reviewers, Microsoft Excel spreadsheets and Microsoft Word documents as needed. We will document all decisions made and rationale surrounding those decisions within these documents.

##### Amendments

Any amendments to this protocol will be documented and reported, with details of amendments and rationale as to why it occurred.

## Discussion

A key strength of the study described within this protocol is the use of a scoping review methodology, which will enable us to follow a transparent and reproducible procedure while being flexible to amendments as we become more familiar with the data. In this protocol, we have described the need for this review, who it will benefit, our target population, setting, search strategy and data we anticipate extracting. Publication of this research protocol is in keeping with good, transparent research practise, as it reduces the risk of bias and selective reporting while providing an opportunity to strengthen our proposed review.

As there are no human participants involved, there will be no requirement for ethical approval. Patients and/or the public were not involved in the design of this protocol; however, the authors will work with patients and members of the public through stakeholder and other PPI research forums in disseminating the findings of the review both in the UK and the Global South.

Findings will be disseminated widely through peer-reviewed publication and in various media, for example, conferences, congresses or symposia. This scoping review will inform other researchers in the field of adolescent health as a standalone piece of work but will also provide a baseline resource which can be used to inform future research planning. To the best of our knowledge, this scoping review is the first attempt to systematically identify and map the alcohol screening and assessments tools used to target adolescent alcohol use in Africa.

## Supplementary Information

**Additional file 1.** PRISMA-P 2015 Checklist

**Additional file 2.** Draft MEDLINE Search Strategy. Results will be limited by English language, date of publication (January 2000 – December 2020) and narrowed by subject age: child (6-12 years), adolescent (13-18 years) and young adult (19-24 years).

**Additional file 3.** Draft CINAHL Search Strategy. Results will be limited by English language, date of publication (January 2000 – December 2020) and narrowed by subject age: child (6-12 years), adolescent (13-18 years) and adult (19-44 years).

**Additional file 4.** Draft Global Health Search Strategy. Results will be limited by date of publication (January 2000 – December 2020). Unable to limit by age in Global Health so fourth “adolescent” concept has been added to the search terms.

**Additional file 5.** Draft Cochrane Search Strategy. Results will be limited by date of publication (January 2000 – December 2020).

## Data Availability

Not applicable

## Abbreviations

AUDIT/AUDIT-C: Alcohol use disorders identification test
HED: Heavy episodic drinking
JBI: Joanna Briggs institute
NGO: Non-governmental Organisations
PCC: Population, concept, context
PICO: Population, intervention, comparator, outcome
PRISMA-P: Preferred reporting items for systematic review and meta-analysis protocols PRISMA-ScR Preferred reporting items for systematic reviews and meta-analyses extension for scoping reviews
WHO: World health Organization
