# Supplementary material for: Adolescent alcohol consumption: protocol for a scoping review of screening and assessment tools used in Africa
Source: Syst Rev. 2021 Apr 8;10:100. doi: 10.1186/s13643-021-01653-1 (PMC8033727; doi:10.1186/s13643-021-01653-1)
Supplement: Supplementary file 2 — Additional file 2. Draft MEDLINE Search Strategy. Results will be limited by English language, date of publication (January 2000 – December 2020) and narrowed by subject age: child (6-12 years), adolescent (13-18 years) and young adult (19-24 years). [file 13643_2021_1653_MOESM2_ESM.docx]

## Additional File 2. Draft MEDLINE Search Strategy

Results will be limited by English language, date of publication (January 2000 – December 2020) and narrowed by subject age: child (6-12 years), adolescent (13-18 years) and young adult (19-24 years).

In July 2020 this search produced 2,117 articles.

| **Concept** | **Search Terms** |
| --- | --- |
| Alcohol | (MH “Alcoholic Beverages+”) OR (MH "Alcohol Drinking+") OR (MH "Alcohol-Related Disorders+") OR (MH "Alcohol-Induced Disorders+") OR (MH "Alcoholism") OR (MH "Underage Drinking") OR (MH "Ethanol+") OR (MH "Binge Drinking") OR (MH "Alcoholic Intoxication") OR alcohol* OR intoxicat* OR drunk |
| Africa | (MH "Africa+") OR Africa* |
| Screening/ assessment tools | (MH "Mass Screening+") OR (MH "Psychometrics") OR (MH "Surveys and Questionnaires+") OR (MH "Health Surveys+") OR (MH "Biomarkers+") OR  assessment* OR screening OR measure* OR biomarker* |
