# Supplementary material for: Adolescent alcohol consumption: protocol for a scoping review of screening and assessment tools used in Africa
Source: Syst Rev. 2021 Apr 8;10:100. doi: 10.1186/s13643-021-01653-1 (PMC8033727; doi:10.1186/s13643-021-01653-1)
Supplement: Supplementary file 4 — Additional file 4. Draft Global Health Search Strategy. Results will be limited by date of publication (January 2000 – December 2020). Unable to limit by age in Global Health so fourth “adolescent” concept has been added to the search terms. [file 13643_2021_1653_MOESM4_ESM.docx]

Additional File 4. Draft Global Health Search Strategy

Results will be limited by date of publication (January 2000 – December 2020). Unable to limit by age in Global Health so fourth “adolescent” concept has been added to the search terms.

In July 2020 this search produced 838 articles.

| **Concept** | **Search Terms** |
| --- | --- |
| Alcohol | (((DE "ethanol") OR (DE "alcoholism")) OR (DE "alcohol intake")) OR (DE "alcoholic beverages" OR DE "beers" OR DE "cider" OR DE "distilled spirits" OR DE "liqueurs" OR DE "mead" OR DE "sake" OR DE "wines") OR alcohol* OR intoxicat* OR drunk |
| Africa | DE "Africa" OR DE "Francophone Africa" OR DE "Africa South of Sahara" OR DE "North Africa" OR DE "Portuguese Speaking Africa" OR DE "Anglophone Africa" OR Africa* |
| Screening/ assessment tools | (((((DE "questionnaires") OR (DE "measurement")) OR (DE "screening")) OR (DE "assessment")) OR (DE "diagnosis" OR DE "laboratory diagnosis" OR DE "diagnostic techniques" OR DE "identification")) OR (DE "surveys" OR DE "surveying"))))) OR assessment* OR screening OR measure* OR biomarker* |
| Adolescents | (((DE "adolescents") OR (DE "young adults")) OR (DE "children" OR DE "boys" OR DE "girls" OR DE "school children")) OR (DE "youth"))) OR adolescen* OR youth* OR "young adult*" OR teen* OR "young person*" OR "young people" OR child* OR juvenile* |
